# Green Tomato Extract Prevents Bone Loss in Ovariectomized Rats, a Model of Osteoporosis

**DOI:** 10.3390/nu12103210

**Published:** 2020-10-21

**Authors:** Farida S. Nirmala, Hyunjung Lee, Ji-Sun Kim, Taeyoul Ha, Chang Hwa Jung, Jiyun Ahn

**Affiliations:** 1 Department of Food Biotechnology, University of Science and Technology, Daejeon 305350, Korea; 50013@kfri.re.kr (F.S.N.); tyhap@kfri.re.kr (T.H.); chjung@kfri.re.kr (C.H.J.); 2 Research Group of Natural Material and Metabolism, Korea Food Research Institute, Wanju 55365, Korea; hjlee1120@kfri.re.kr (H.L.); jskim320@gmail.com (J.-S.K.); 3 Department of Biotechnology, College of Life Science and Biotechnology, Korea University, Seoul 02841, Korea

**Keywords:** nutritional supplement, postmenopausal osteoporosis, bone homeostasis, green tomatoes

## Abstract

Although drug therapies are available for postmenopausal osteoporosis, these drugs are not free of side effects and long-term adherence to them are low. A safe and effective nutritional approach to counter postmenopausal osteoporosis is an important research goal. We fed ovariectomized (OVX) Sprague–Dawley rats a diet supplemented with 1% or 2% green tomato extract (GTE). After 12 weeks, micro-computed tomography scans revealed that GTE supplementation effectively prevented distal femur bone loss. This prevention was due to improved bone formation and suppressed bone resorption as observed by the regulation of osteoblast and osteoclast activities. GTE supplementation also improved bone formation through Bmp2-Smad 1/5/8-Runx2 signaling, while bone resorption was regulated by the receptor activator of nuclear factor kappa-B (RANKL)/osteoprogeterin (OPG) pathway. These results suggest that GTE supplementation prevents severe postmenopausal bone loss by maintaining the regulation of bone homeostasis in OVX rats. GTE as a diet supplement might be a potential novel alternative for the prevention of postmenopausal osteoporosis.

## 1. Introduction

Bone metabolism mainly relies on the dynamic equilibrium of osteoblasts and osteoclasts, given that these cells are crucial in maintaining homeostasis [1]. Homeostasis of bone metabolism is very complex and involves aging, secretion of hormones, calcium malabsorption, disease related damages, and nutrition. Disrupted bone homeostasis may result in pathological conditions, such as osteoporosis [2,3,4,5]. 

Osteoporosis comprises primary and secondary forms. Primary osteoporosis includes postmenopausal osteoporosis, which is specific to females [5]. Postmenopausal osteoporosis is a bone degenerative disease characterized by extensive loss of bone mass and significant disruption of bone mechanical properties. It directly results in extreme bone fragility and vulnerability to fractures [6]. Estrogen deficiency in menopause interrupts bone homeostasis in which osteoblasts fail to perform bone formation to cope with elevated osteoclasts activity [7,8]. Continued disruption of bone homeostasis elevates the risk of postmenopausal women for worsened quality of life. Postmenopausal osteoporosis is the reason for the statistical dominance of female osteoporosis patients [9]. Considering its risks, prevention of postmenopausal osteoporosis is an actively pursued research goal. 

Current available options on osteoporosis treatment are still far from optimal [10,11,12]. Identification of agents that helps bone loss prevention would have substantial effect on the development of treatments for osteoporosis. Earlier studies have proposed the possibility of a nutritional approach as an alternative for the prevention of postmenopausal osteoporosis [13,14,15,16,17,18]. 

Tomatoes, which belong to the family Solanaceace, are one of the most commonly consumed vegetables [19]. Tomatoes are nutritious and contain many bioactive compounds, yet its effect on specific diseases is still widely unknown. Among various nutrients in tomatoes, tomatidine, aglycone of a-tomatine, is present as a major glycoalkaloid especially abundant in unripe tomato fruits [20]. Green tomatoes have a high content of tomatine up to 500 mg/kg, while ripe tomatoes have less than 5 mg/kg [20]. Tomatidine has attracted interest for its many biologic activities, which include cardioprotective, anti-inflammatory, antioxidative, and lifespan extension [20,21,22].

Here we show that the whole green tomato extract (GTE) potentially prevents postmenopausal osteoporosis in ovariectomized (OVX) rats. GTE promotes osteoblast activity through bone morphogenetic protein 2 (Bmp2)-Smads 1/5/8-Runx2 signaling and suppresses osteoclast activity by regulating receptor activator of nuclear factor kappa-Β ligand (RANKL)-RANK binding and NADPH oxidase 4 (Nox-4) expression. The findings of the study suggest that GTE might be a potential alternative treatment for postmenopausal osteoporosis.

## 2. Materials and Methods 

### 2.1. Preparation of Samples

Dried red and green tomatoes (Korean *chal* tomatoes, 0.5 g) were ultra-sonicated with 2 mL of 5% acetic acid for 30 min, followed by stirring for 2 h and another sonication (30 min). During this process, the temperature was maintained under 40 °C to avoid degradation of sample constituents. The suspension was then centrifuged for 5 min at 12,000 rpm and filtered through a 0.45 µm pore size membrane. The extract was further purified using a solid phase extraction (SPE) column (Discovery® DSC-SCX; Supelco, Bellefonte, PA, USA). The SPE column was pre-conditioned with 10 mL of methanol and 10 mL of 5% acetic acid. The extraction was applied and allowed to gravity-drip. When the sample was fully absorbed onto the packing, the tube was washed with 20 mL of 5% methanol. The tomatidine was eluted with 20 mL of 2.5% NH3 in methanol. The purified extracts were evaporated to dryness under reduced pressure under 40 °C, re-dissolved in methanol, and filtered through a 0.45 µm pore size membrane.

### 2.2. Tomatidine Analysis by High-Performance Liquid Chromatography (HPLC)

Tomatidine was purchased from Sigma-Aldrich (St. Louis, MO, USA). Standard stock solution was prepared at a concentration of 2 mg/mL in 80% methanol. The standard solution was serially diluted with 80% methanol to obtain calibration standard solutions at concentrations of 6.25, 12.5, 25, 50, 100, and 200 µg/mL. An HPLC apparatus equipped with a PU-2089 pump, UV-2075 spectrophotometer, and CO-2065 column oven (Jasco, Tokyo, Japan) was used. Chromatographic separation was achieved using an XTerra RP 18 column (4.6 × 150 mm, 5 µm; Waters, Milford, MA, USA) with the column oven temperature maintained at 35 °C. The mobile phase consisted of acetonitrile (Solvent A) and 25 mM triethylammonium phosphate in water (pH 3.0; Solvent B). The mobile phase flow rate was 0.8 mL/min with gradient elution. The initial percentage composition of Solvent A was 20%. It was gradually increased to 45% for 12 min, to 55% for 5 min, and to 57% for 3 min, followed by equilibration to the initial composition for 5 min. All solvents for HPLC analysis were of HPLC grade and were purchased from J.T. Baker (Phillipsburg, NJ, USA). 

### 2.3. Animal Works

A total of fifty 7-week old Sprague–Dawley (SD) rats were obtained from Japan SLC. Inc (Hamamatsu, Japan). After one week of acclimatization, ten rats were sham operated (Sham) and fed with the AIN-93G diet. Forty rats were subjected to ovariectomy surgery and divided into four different groups of ten animals each. The control (Con) group was fed the AIN-93G diet. Experimental groups were fed the AIN-93G diet containing 0.04% estrogen (E2), 1% GTE (low dosage group, GL), or 2% GTE (high dosage group, GH). The experimental diet was fed for 12 weeks, starting at age of 13-weeks old. Body weights were measured weekly. Upon sacrifice, femur and tibia bones were extracted and their masses were measured. Relative bone mass was determined by dividing the weight of bone by the total body weight. The experimental protocol was approved by the Korea Food Research Institute Animal Care and Use Committee (KFRI-IACUC, #M15-0025).

### 2.4. Micro-Computed Tomography (Micro-CT) Analysis

Distal metaphysis of the tibia was analyzed using the SkyScan 1076 micro-CT scanning system (Bruker Micro-CT, Seeshaupt, Germany) at a resolution of approximately 18 μm/μm^3^. Morphometric parameters including bone mass density (BMD), bone volume fraction (BV/TV), bone surface ratio (BS/TV), and trabecular thickness (Tb.Th) were individually measured using software package provided by the instrument.

### 2.5. Serum and Urine Biochemical Assays

Serum was collected at the end of the experiment then used for measurements of aspartate aminotransferase (AST) and alanine aminotransferase (ALT) as instructed by the manufacturer (Ire Medical, Seoul, Korea). Serum adiponectin (RnD System, USA) and leptin (ALPCO, NH, USA) were measured as instructed by the manufacturers. Bone-specific alkaline phosphatase (bALP), total alkaline phosphatase (tALP), rat RANKL, and rat tartrate resistant acid phosphatase 5b (TRACP5B) were measured according to the instructions provided by the manufacturer (Kamiya Biomedical Co., Seattle, WA, USA). For urine collection, rats were placed in metabolic cages for 16 h at the end of the animal experiment. Urine samples were stored at −80 °C until analysis. Urinary calcium (Cayman Chemical, Ann Arbor, MI, USA) and deoxypyridinoline (DPD; Kamiya Biomedical Co., Seattle, WA, USA) were measured according to the manufacturer’s instructions.

### 2.6. Quantitative Real-Time PCR (qRT-PCR) Analysis

Total RNA from the tibia was isolated using TRIzol reagent (Invitrogen Life Technologies, Carlsbad, CA, USA) and a NucleoSpin RNA II Kit (Macherey-Nagel GmbH & Co., Duren, Germany) according to the instructions provided by the manufacturers. To measure mRNA expression, cDNAs were prepared as previously described [20], and qRT-PCR was performed using the SYBR Green PCR Master Mix in the StepOnePlus Real-Time PCR system (Applied Biosystems, Foster City, CA, USA). The expression level of each mRNA was normalized to that of β-actin. The primer sequences used for the experiment are shown in Table 1. 

### 2.7. Statistical Analysis

Data are expressed as means ± standard error of the mean (SEM). One-way ANOVA was used to compare the quantitative data among groups using GraphPad Prism 8 (San Diego, CA, USA). The Dunnet post-hoc test was used when ANOVA results were significant (*p* < 0.05). 

## 3. Results

### 3.1. Green Tomatoes Contain a Higher Content of Tomatidine than Ripened Red Tomatoes 

**Figure 1:**
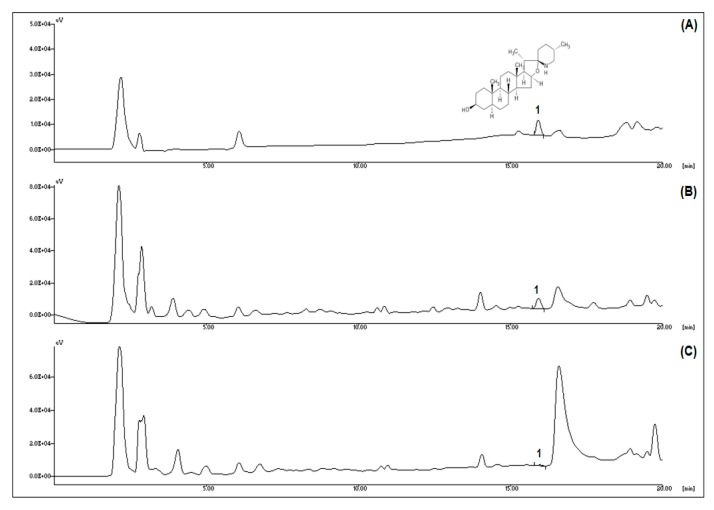
Tomatidine profiles of green and red tomatoes. (**A**) Chromatogram of typical tomatidine. (**B**) Chromatogram of green tomatoes. (**C**) Chromatogram of red tomatoes. Peak identification: 1, tomatidine.

HPLC analysis was performed to determine the tomatidine content during ripening (Figure 1). The typical chromatogram of tomatidine is shown in Figure 1A. Compared to green tomatoes, the tomatidine peak was markedly decreased in red tomatoes (Figure 1B,C). As shown in Table 2, the tomatidine content of green tomatoes (1.06 ± 0.11 mg/100 g dry weight) was significantly higher than in red tomatoes (0.13 ± 0.01 mg/100 g dry weight). The data confirmed that the ripening leads to decreased tomatidine content in tomatoes. Therefore, green tomatoes are a better source of tomatidine than red tomatoes.

### 3.2. GTE Prevents Weight Gain and Increases Bone Mass in OVX Rats

**Figure 2:**
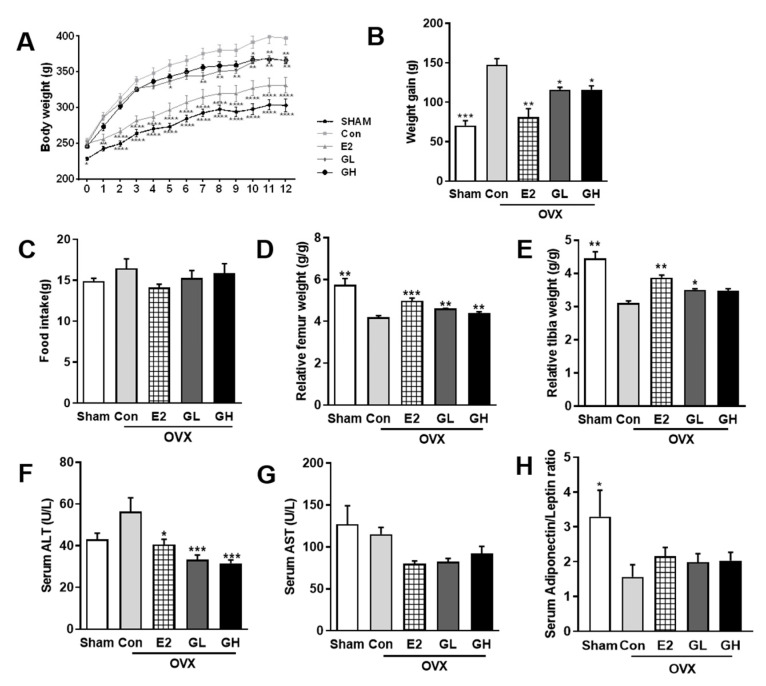
Body and bone phenotype changes in green tomato extract (GTE) supplemented ovariectomized (OVX) rats. (**A**) Weekly body weight measurement during experimental period. (**B**) Total weight gain of OVX rats over 12 weeks. (**C**) Average food intake of 12 weeks. (**D**) Femur weight relative to the total body weight. (**E**) Tibia weight relative to the total body weight. (**F**) Serum aminotransferase (ALT) content. (**G**) Serum alanine aminotransferase (AST) content. (**H**) Serum adiponectin/leptin ratio. Results are expressed as means ± SEM. Con, control. *** *p* < 0.001, ** *p* < 0.01, * *p* < 0.05, significantly different from Con.

Next, we measured the effect of GTE on OVX-evoked bone loss. For this, we supplemented rats with 1% or 2% GTE (GL and GH, respectively) for 12 weeks. Induction of postmenopausal condition by OVX surgery resulted in dramatic weight gain in the control group compared to the sham operated group (Figure 2A,B) despite no significant difference was found in the food intake of all groups (Figure 2C). Supplementation with GTE successfully lowered postmenopausal weight gain. In accordance with the lowered body weight, both liver and white adipose tissue weights were also significantly decreased in GTE supplemented groups (Table 3). In addition, measurements of serum aminotransferase (AST) and alanine aminotransferase (ALT) contents suggested that GTE supplementations did not induce liver toxicity (Figure 2E,F). We also observed slight improvement of serum adiponectin/leptin ratio in GTE supplemented groups, indicating that GTE might regulate the adipogenic metabolism of OVX rats although the role is minor (Figure 2G). Most importantly, the relative femur and tibia weights were significantly increased in GTE supplemented groups, compared to the control group (Figure 2C,D). These data indicated that supplementation of GTE ameliorated body and bone phenotype in the postmenopausal rat model. 

### 3.3. GTE Increases BMD in OVX Rats

**Figure 3:**
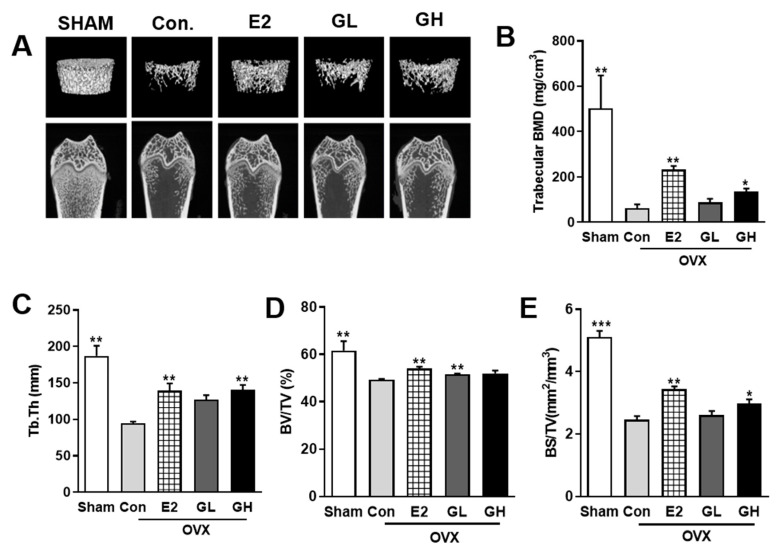
Effect of GTE supplementation on bone loss in OVX rats. (**A**) Representative two- and three-dimensional micro-CT images of trabecular bones in the distal femurs of rats from each group. (**B**) Measurement of trabecular bone mass density (BMD) in the distal femurs. Measurement of the trabecular morphometric parameters of trabecular thickness (Tb.Th) (**C**), bone volume/total volume (BV/TV) (**D**), and bone surface/total volume (BS/TV) (**E**) in the distal femurs. Results are expressed as means ± SEM. *** *p* < 0.001, ** *p* < 0.01, * *p* < 0.05, significantly different from Con.

To investigate the effect of GTE supplementation on the prevention of postmenopausal bone loss, we performed micro-CT analysis of femurs (Figure 3A). Both two-dimensional and three-dimensional micro-CT images showed distinct loss of trabecular bone in control group compared to the sham operated group. Estrogen treatment as a positive control (E2) successfully defended femur against bone loss in OVX rats. Interestingly, GTE supplementation groups exhibited better density of femur, indicating successful prevention against OVX-evoked bone loss. Furthermore, quantification of trabecular BMD showed that the protective effect of GTE against bone loss was particularly significant in higher dose (GH) (Figure 3B). Compared to the control group, several trabecular parameters, including Tb.Th, BV/TV, and BS/TV, were also increased by GTE supplementation (Figure 3C–E). Taken together, these results indicated that GTE supplementation improved BMD and overall bone quality in OVX rats.

### 3.4. GTE Improves Bone Formation in OVX Rats

**Figure 4:**
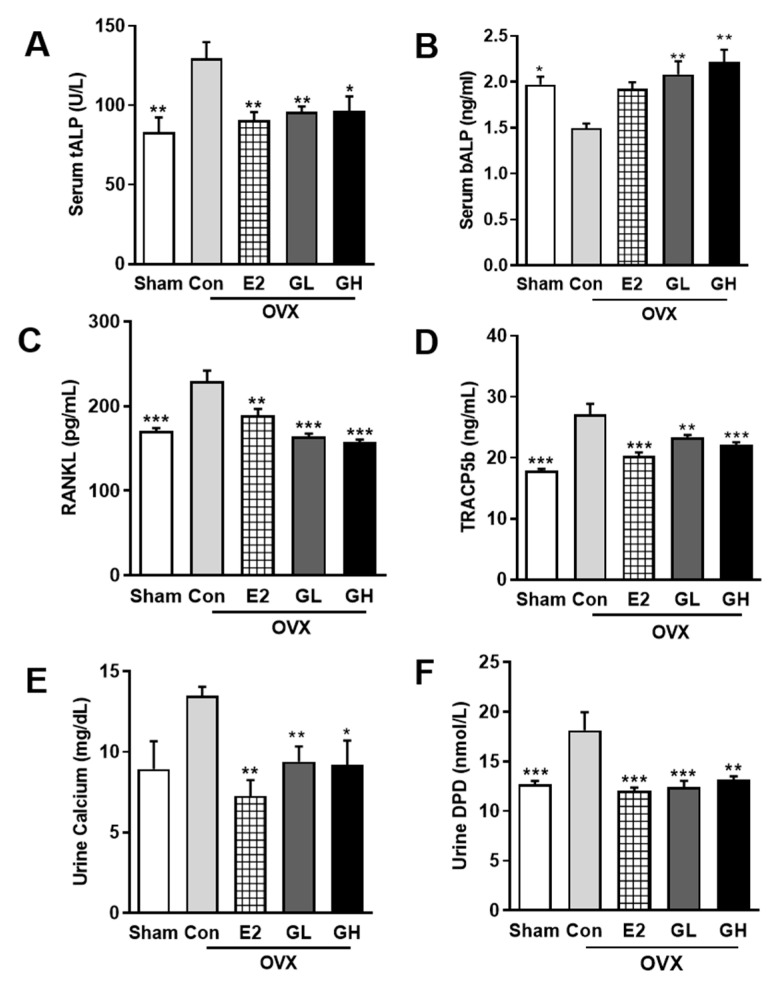
Effect of GTE supplementation on markers of osteoblasts and osteoclasts in serum and urine. Measurements of serum total alkaline phosphatase (tALP) (**A**), bone alkaline phosphatase (bALP) (**B**), receptor activator of nuclear factor kappa-B (RANKL) (**C**), and tartrate-resistant acid phosphatase 5b (TRACP5b) (**D**). Measurements of urine calcium (**E**) and deoxypyridinoline (DPD) (**F**). Results are expressed as means ± SEM. *** *p* < 0.001, ** *p* < 0.01, * *p* < 0.05, significantly different from Con.

Hypothetically, increased BMD in OVX rats by GTE supplementation should be followed with improved bone formation and lowered bone resorption. To prove this hypothesis, we examined several markers of bone formation and resorption. The primary bone formation marker, serum ALP, was profoundly altered in the OVX control group compared to the sham operated group (Figure 4A,B). In serum, total ALP content was significantly increased, while bone specific ALP was reduced, indicating the inability of bone formation in the OVX control group. However, GTE supplementation effectively recovered the altered total and bone specific ALP levels, suggesting the effect of GTE on increasing bone formation. By contrast, serum levels of RANKL and TRACP5b were significantly increased in the control group compared to the sham operated group. This increase was evident as higher bone resorption activity in the control group. GTE supplementation successfully reduced serum levels of RANKL and TRACP5b (Figure 4C,D). Hence, GTE supplementation potency in suppressing bone resorption was observed. Furthermore, reduced calcium absorption in OVX rats was significantly prevented by GTE supplementations, as showed by urine calcium analysis results (Figure 4E). Lowered urinary DPD level was also observed in GTE supplemented groups (Figure 4F). Collectively, these results indicated that GTE supplementation prevented postmenopausal bone loss by improving bone formation and lowering bone resorption activity in OVX rats.

### 3.5. GTE Increases Bone Formation by Regulating Bmp2-Smads 1/5/8-Runx2 Signaling

**Figure 5:**
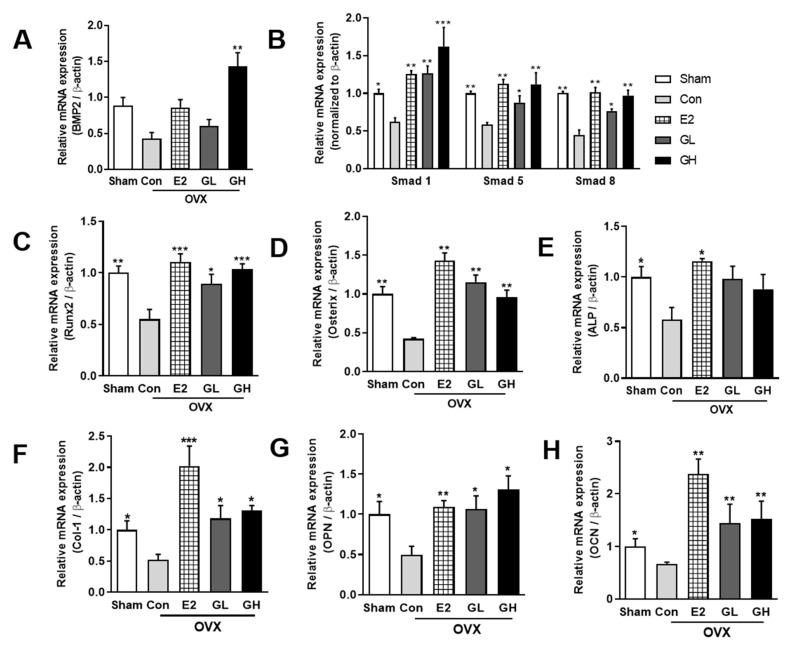
Effect of GTE supplementation on bone formation-related signaling genes. The relative mRNA expression of BMP2 (**A**), Smad 1/5/8 (**B**), Runx2 (**C**), Osterix (**D**), alkaline phosphatase (ALP) (**E**), Collagen 1 (Col-1) (**F**), osteopontin (OPN) (**G**), and osteocalcin (OCN) (H) in tibia bones of all groups. Results are expressed as means ± SEM. *** *p* < 0.001, ** *p* < 0.01, * *p* < 0.05, significantly different from Con.

To determine the underlying mechanism of bone formation improvement by GTE supplementation, we measured the mRNA expressions of several bone formation-related genes using qRT-PCR. The expression of Bmp2, a representative bone formation signaling molecule, was significantly downregulated in the OVX control group compared to the sham operated group (Figure 5A). GTE supplementation in OVX rats upregulated Bmp2 mRNA expression, especially up to ±3.1-fold in the GH group. We then analyzed the downstream signaling markers of Bmp2. The expressions of Smads 1/5/8 genes were notably downregulated in the control group compared to the sham operated group (Figure 5B). Contrary to the control group, GTE supplemented groups showed significantly upregulated Smads 1/5/8 mRNA levels in a dose-dependent manner. The markedly decreased Runx2 and Osterix in OVX rats were significantly upregulated by GTE supplementation (Figure 5C,D). Finally, the mRNA expressions of the bone formation markers ALP, collagen type 1 alpha 1 chain (Col1α1), osteopontin (OPN), and osteocalcin (OCN) were all downregulated in the control group compared to the sham operated group (Figure 5E–H). Treatment of GTE induced significant increases of the bone formation markers. Collectively, these data demonstrated that increased bone formation by GTE supplementation was regulated by Bmp2-Smads 1/5/8-Runx2 signaling. 

### 3.6. GTE Decreases Bone Resorption through the RANKL/OPG Pathway

**Figure 6:**
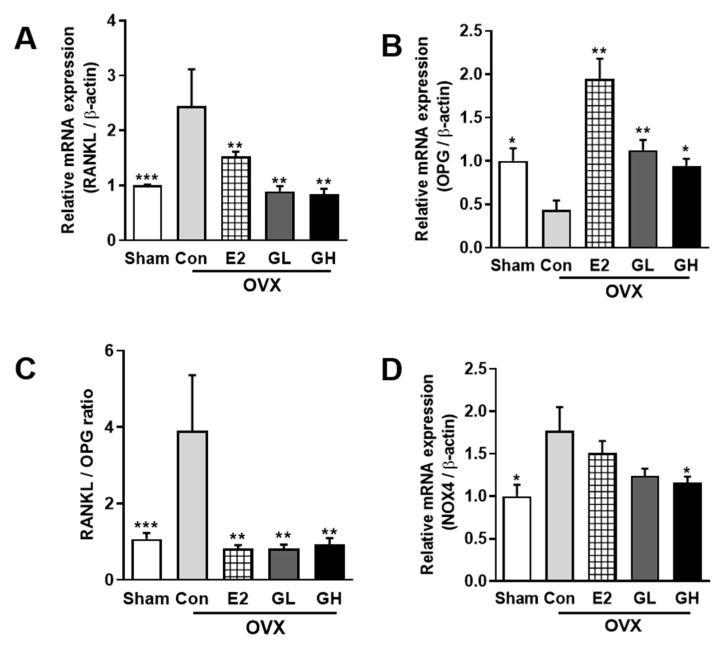
Effect of GTE supplementation on osteoclast activities. The relative mRNA expression of RANKL (**A**) and OPG (**B**) measured using quantitative RT-PCR in tibia bones of all groups. (**C**) Calculation of RANK/OPG ratio. (**D**) Relative mRNA expression of Nox-4. *** *p* < 0.001, ** *p* < 0.01, * *p* < 0.05, significantly different from Con.

To elucidate the effect of GTE supplementation on bone homeostasis, we analyzed the bone resorption-regulated genes by qRT-PCR. First, we found that mRNA expression of RANKL, an osteoclastogenesis activator, was significantly upregulated in the control group compared to the sham operated group (Figure 6A). In contrast, mRNA expression of osteoclastogenesis inhibitor gene, osteoprotegerin (OPG), was significantly downregulated in the control group compared to the sham operated group (Figure 6B). We then calculated the RANKL/OPG ratio and found that the ratio was remarkably elevated in control group compared to the sham operated group, suggesting a higher rate of RANKL binding in the control group (Figure 6C). GTE supplementation improved the dysregulated RANKL and OPG mRNA levels and recovered the RANKL/OPG ratio. Moreover, the upregulation of Nox-4 by OVX was decreased by GTE supplementation, although a significant difference was only observed in GH group (Figure 6D). These data demonstrated that GTE supplementation regulated the osteoclastogenesis related gene at the mRNA level and, thus, prevented bone resorption in OVX rats.

## 4. Discussion

The nutritional approach as an alternative treatment for postmenopausal osteoporosis treatment has attracted interest of many researchers. A recent study proposed that tomatidine can prevent bone loss by suppressing osteoclast activity [18]. Tomatoes are the most prevalent tomatidine source. Problematically, tomatoes reportedly decline in tomatidine content during ripening [21]. Most importantly, suppression of osteoclast activity alone might not be the most optimal option to prevent postmenopausal osteoporosis, as its prevention does not rely exclusively on osteoclast activity, but rather is more dependent on bone metabolism equilibrium in which osteoblasts play crucial roles as well [22]. 

In this study, we confirmed that green tomatoes contain a higher content of tomatidine than red tomatoes. We also examined the effect of green tomatoes with high tomatidine on postmenopausal osteoporosis using two dosages of GTE in the diet consumed by OVX rats. GTE supplementation improved BMD in OVX rats. According to these results, green tomatoes might be a potential alternative for tomatidine-induced prevention of postmenopausal osteoporosis. 

**Figure 7:**
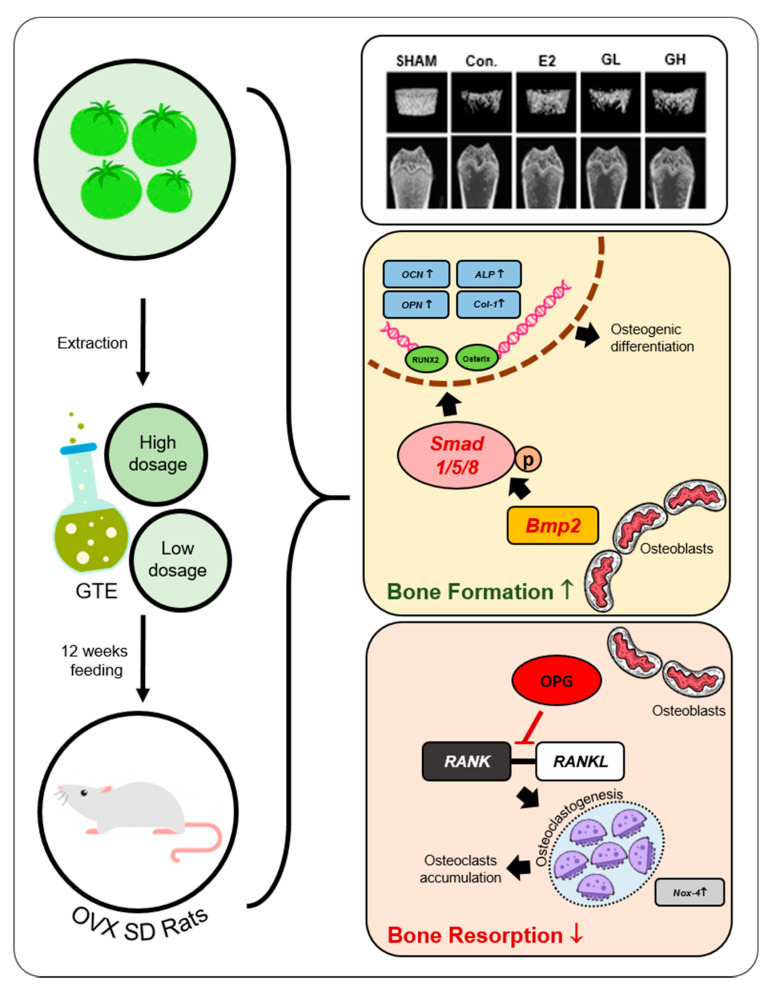
Schematic diagram for the effect GTE supplementation on the prevention of postmenopausal osteoporosis.

The defensive mechanism against postmenopausal bone loss involves osteoblast activity in regulating complex signaling pathways to orchestrate bone formation. Bmp2, a member of the transforming growth factor-beta superfamily, is predominantly synthesized and secreted by osteoblasts [23]. Bmp2 is crucial in osteogenesis and abrogation of its function impairs bone growth [24,25,26]. Bmp2 promotes osteogenesis by regulating the expression of Runx2 and Osterix, the key transcription factors involved in osteoblast differentiation [27,28]. We analyzed Bmp2 mRNA expression and found that GTE supplementation, especially high-dose group (GH), significantly upregulated Bmp2 and its downstream genes. It is suggested that, in the models used in this study, Bmp2 regulation might lean towards Runx2 rather than Osterix as projected in mRNA expressions. Nevertheless, the Bmp2 upregulation resulted in higher trabecular BMD, suggesting that Bmp2 might be responsible for the improvement of bone mass in GTE supplemented OVX rats (Figure 7).

Aside from bone formation, bone resorption is a part of the bone homeostasis, which is entirely influenced by the activity of osteoclasts [29]. Osteoclastogenesis plays crucial role in the regulation of bone resorption activity as it multinucleates and resorbs bone [30]. Our assessment of the alteration of osteoclastogenesis related genes showed that GTE supplementation remarkably decreased the ratio of RANKL/OPG mRNA expression. RANKL binds to RANK to complete osteoclastogenesis. When osteoclastogenesis increases, osteoblasts produce OPG to inhibit the binding process between RANKL and RANK [31,32]. Balance of this systematic interaction between osteoclasts and osteoblasts is crucial in maintaining bone homeostasis for healthier bones. Therefore, the observed decrease of the RANKL/OPG ratio suggested that GTE supplementation potentially obstructed osteoclastogenesis by regulating OPG in the inhibition of RANKL binding. Interestingly, Nox-4 expression was also downregulated by the supplementation of GTE. Nox-4 is a notable gene induced during the osteoclastogenesis process. The loss of Nox-4 activity halts the osteoclastogenesis process and favors the bone formation process [33,34]. The present observation of lowered Nox-4 expression demonstrated that the total osteoclastogenesis process was suspended in GTE supplemented OVX rats.

Collectively, our results suggest that GTE precludes excessive bone loss due to postmenopausal osteoporosis by inducing bone formation through Bmp2-Smads 1/5/8-Runx2 signaling and interrupts osteoclastogenesis via RANKL/OPG binding and lowered Nox-4 activity (Figure 6E). However, considering that we utilized growing rats in this study, further study regarding the implementation of GTE supplementation in adult postmenopausal osteoporosis prevention is needed in the future. Nevertheless, our findings offer a promising possibility of GTE as a novel nutritional approach alternative to prevent postmenopausal osteoporosis through the regulation of bone homeostasis.

## Figures and Tables

**Table 1:** Primer sequences.

Gene.	Sequence (5′→3′)
BMP2	F: TGA GGA TTA GCA GGT CTT TG
	R: CAC AAC CAT GTC CTG ATA AT
Smad1	F: TCAATAGAGGAGATGTTCAAGCAGT
	R: AAACCATCCACCAACACGCT
Smad5	F: CCCTATCCTCCTTCCCCTGC
	R: GAGGGGTATCAGCTGGGAGTT
Smad8	F: ACT TCC GGC CAG TTT GCT AC
	R: TGG GGA TCT TGC AGA CAG TG
Runx2	F: GGC GTC AAA CAG CCT CTT CA
	R: GCT CGG ATC CCA AAA GAA GTT
Osterix	F: TGG CCA TGC TGA CTG CAG CC
	R: TGG GTA GGC GTC CCC CAT GG
ALP	F:GCACAACATCAAGGACATCG
	R:TGGCCTTCTCATCCAGTTCA
Col-1	F: GCA TGG CCA AGA AGA CAT CC
	R: CCT CGG GTT TCC ACG TCT C
OPN	F: GAT GAA TCT GAC GAA TCT CAC C
	R: CTC AGA AGC TGG GCA ACA GGG AT
OCN	F: CAG ACA AGT CCC ACA CAG
Rankl	F:TGAAGACACACTACCTGACTCCTG
	R:CCACAATGTGTTGCAGTTCC
OPG	F:GTTTCCCGAAGGACCACAAT
	R:CCATTCAATGATGTCCAGGAG
NOX4	F: CTG CAT CTG TCC TGA ACC TCA A
	R: TCT CCT GCT AGG GAC CTT CTG T
β-actin	F:TCTTCCAGCCTTCCTTCCTG
	R:TAGAGCCACCAATCCACACA

**Table 2:** Tomatidine contents of tomato fruits.

Tomato Samples	Tomatidine Content (mg/100 g Dry Weight)	*p*-Value
Green	1.06 ± 0.11	*p* < 0.001
Red	0.13 ± 0.01	

Results are expressed as mean ± standard deviation of triplicate.

**Table 3:** Rats organelle weight.

Weight (g)	Sham	Con	E2	GL	GH
Liver	9.76 ± 0.34	10.48 ± 0.18	8.30 ± 0.35 **	8.91 ± 0.24 ***	8.20 ± 0.28 ***
WAT (Retroperitoneal)	11.03 ± 0.04 **	17.94 ± 0.33	8.12 ± 0.99 ***	12.98 ± 0.73 **	12.89 ± 0.93 **
Spleen	0.58 ± 0.02	0.63 ± 0.02	0.58 ± 0.02	0.59 ± 0.01	0.61 ± 0.02
Kidney	1.74 ± 0.08	1.66 ± 0.04	1.64 ± 0.05	1.64 ± 0.04	1.63 ± 0.04

Results are expressed as mean ± SEM with *** *p* < 0.001, ** *p* < 0.01. Sham: sham operated group; Con: OVX operated group; E2: OVX operated + estrogen; GL: OVX operated + GTE low dosage; GH: OVX operated + GTE high dosage.
